# The Medical Association of Georgia

**Published:** 1896-05

**Authors:** 


### *SOCIETY REPORTS.*

### THE MEDICAL ASSOCIATION OF GEORGIA.

AUGUSTA, April 15, 16, and 17, 1896.

The Forty-Seventh Annual Meeting of the Association met in the Richmond county courthouse, and was called to order by the President, Dr. Frank M. Ridley, of LaGrange.

The "Address of Welcome" was delivered by Dr. Eugene Foster, of Augusta, which was responded to by Dr. E. R. Anthony, of Griffin.

President Ridley then delivered his

ANNUAL ADDRESS, in which he forcibly and eloquently dwelt upon several topics of interest to the profession.

The first paper read was by Dr. W. J. Mathews, of Middleton, entitled "The Treatment of Pneumonia."

The author had never seen a case in his experience where he thought the symptoms warranted abortive treatment, owing to not seeing the patient until a few hours after the prodromic chill, the lung then being engorged to such an extent that he thought his efforts would prove futile. Having satisfied himself that he has a case of pneumonia to deal with, his first step is to make the patient comfortable. To meet this end he envelops the affected side with counter-irritation, preferably turpentine. To relieve restlessness, pyrexia and pain he gives phenacetin. Should phenacetin fail, he gives morphine hypodermically. To reduce hyperpyrexia he administers phenacetin, in sufficient doses, every three or four hours in connection with a stimulant, the purpose being to reduce the temperature to 101° or 102°. In his experience phenacetin does not, like some of the other coal-tar products, depress the heart. He advocates the application of blisters. He had never seen a case calling for venesection. To control the pulse he gives veratrum viride every three or four hours with whisky. The overworked heart should be closely watched, its action sustained, and clot prevented. This he does by digitalis and carbonate of ammonia. In the stage of resolution he resorts to supportive treatment. The type of which he had spoken was the acute lobar variety.

Dr. J. W. Duncan, of Atlanta, seldom blisters an adult. In the very initial stage a blister might do some good. He does not give the coal-tar preparations in pneumonia, for the reason that if they are given to reduce temperature they are liable to produce cyanosis. He frequently uses veratrum viride, and considers it a sheet-anchor in this disease.

"Colles's Fracture" was the title of a paper read by Dr. J. B. Morgan, of Augusta. The most common and frequent fractures with which the general practitioner had to do was a Colles fracture. The diagnosis of this fracture is not difficult. The history of the accident, the characteristic deformity, pain and seat of the lesion point at once to the nature of the fracture. Every case of Colles's fracture can be readily reduced by strong, forced dorsal flexion. This is best done under an anesthetic. The best temporary permanent dressing is Wyeth's modification of Pilcher's. The plaster of Paris dressing is an excellent one, and is to be preferred in old people where there is firm impaction which the surgeon does not care to break up, or in cases where the fragments are more or less comminuted. It should be applied from the lower border of the metacarpus to the middle third of the forearm, with the patient's hand in the straight position. A straight dorsal splint may also be employed, but it is not as desirable as the plaster of Paris. Under no circumstances use the angular, crooked, or pistol-shaped splint, and no form of splint should be allowed to project beyond the metacarpus. The fingers must remain freely movable to prevent stiffness of the joint. Limited motion should be encouraged at first; later, active and free. In aged patients, where we have more or less impaction of the broken ends, reduction should not be attempted, as it is better to have a crooked deformed wrist than a failure in bony union, and impaction favors the consolidation of the fractured bone.

Dr. W. L. Champion, of Atlanta, contributed a paper on "The

Importance of Careful Chemical and Microscopical Examination of Urine in Applicants for Life Insurance." Within the past few weeks the author had examined patients with kidney lesions, who a few days or weeks previously had applied for life insurance and had been recommended as good risks. Every medical examiner, after due deliberation, ought to be able, after making a thorough physical examination, having considered the applicant's predisposition to any disease, and last but not least, having made a thorough chemical and, if necessary, microscopical examination of the urine, tell the life expectancy of the applicant before him. Cases were cited by the author to demonstrate that a great many practitioners are too prone to overlook the condition of the kidneys, not to make a careful and thorough examination of the urine, not only in examining applicants for insurance, but their desire to formulate a correct diagnosis in diseased conditions should make it so. He urges the extension of chemical and microscopical investigations in this line of work.

Dr. A. W. Stirling, of Atlanta, in the discussion, called attention to some experiments which he had made some years ago, and which formed the basis of a graduation thesis to the University of Edinburgh. Chiefly through the observations of Pavy, Johnson, and others, it was becoming known that every phase of albuminuria was not a cause of Bright's disease. In order to ascertain the actual number of persons in whom albuminuria was present in health, he examined 369 boys between the ages of twelve and sixteen, who belonged to one of the large training ships on the Thames, near London. In order not to make a mistake in regard to the presence of albumin, he examined each one with four different reagents. He found albumin most common three hours after the boys got out of bed and assumed the erect posture, when the percentage was by examination 20.8 per cent. Of the boys that played in a band the percentage of albumin was about 60 per cent., while in those that did not play in the band it was only 12.8 per cent. Mr. Stichel, of Paris, had seen blindness produced by the playing of wind instruments—due, he says, to passive cerebral congestion with a similar condition of the retina and choroid.

Dr. Stirling says that the urine is usually found to be absolutely free from albumin while the patient remains in bed, and it makes its appearance within a variable but generally short time after the erect posture, and independently of food and all other conditions. The albumin continues in the urine for only an hour or two, or perhaps in varying quantity throughout the day, but as a rule it is disappearing in the afternoon, and is entirely gone by bedtime. The ingestion of food has little effect in producing the albumin, and it is not breakfast which causes its frequency of appearance in the morning. Sometimes he had seen a slight rise after a meal, but this is trivial and by no means constant. He had boys brought ashore to the infirmary and confined to bed, and then albumin was absent from the urine until they got up, breakfast or other meals having no effect. He had made very many and various experiments in connection with this subject, all tending to prove the same thing.

He examined 92 other cases, whose ages varied from 5 to 94, with the result that there was an increase in the percentage of people having albumin with advancing years. From 20 to 30 years he found the percentage of albumin to be only 10 per cent.; 30 to 40, 25 per cent.; 40 to 50, 36.4 per cent.; 50 to 60, 66.6 per cent.; 60 to 70, 75 per cent.; 70 to 80, 75 per cent.; 80 to 90, 83 per cent. These observations were confirmed by Grainger Stewart.

With regard to the advisability of insuring cases of cyclic albuminuria, he would suggest that in the present state of our knowledge, the most satisfactory method of dealing would be for the companies to accept an apparently healthy albuminuric at a high premium, agreeing to rearrange this on their being satisfied of the disappearance of the albumin for such a length of time as would give reasonable expectation of this being permanent.

Dr. Bernard Wolff, of Atlanta, read a paper on "The Modern Treatment of Skin Diseases." See this issue.

Dr. M. B. Hutchins, of Atlanta, said the modern treatment of skin diseases should embrace internal as well as local or external treatment. The patient's constitution should not be overlooked, although if he had to choose between local and internal treatment, he should prefer the former in the treatment of 99 per cent. of the cases of affections of the skin. He had not given the plaster mulls of Unna much trial. They had been objected to on account of containing a gutta-percha base, and in any event the dermatologist would have to carefully select the cases in which to use them. With reference to soaps, such as the bichloride of mercury, the chemical change which takes place renders the remedy inert, and it is almost impossible to get any absorption through the unbroken epidermis. Success in the treatment of syphilis by mercurial inunctions is attained, not by the absorption of the mercury through the skin, but by inhalation of the vapors from the mercurial ointment.

As regards gelatin paste, he had used it a little, but thought it was objectionable because it stuck to everything with which it came in contact, particulaly woolen goods.

In some cases of acne on the faces of young ladies, he had obtained good results by using the constant galvanic current to the cheeks where the eruption appeared. The stimulating action which takes place encourages the glands to throw off secretions without allowing them to remain sluggish and thus block up the follicles of the skin.

A paper on "Appendicitis" was read by Dr. Samuel Loyd, of New York City. It was largely statistical. In making his compilation the author began with the earliest recorded cases, and it covers a greater number of medical than surgical cases. The total number examined up to April 5th, 1896, was 558, and the result was that 263 recovered, and 295 died. Of this number 226 were operated upon; 192 recovered and 31 died. On the other hand, in direct contrast to this, 265 cases were treated conservatively; 60 recovered, while 205 died.

A case was cited which the author had seen in consultation, illustrating the course of extraperitoneal abscesses.

According to the table of cases that the author had examined, the pathological conditions could be summed up as follows:

1. Cases in which the disease follows traumatism.

2. Cases following septic inflammations in other parts of the body, such as salpingitis in the female, post-partum septic inflammation of the uterus, typhoid fever, tuberculosis.

3. Direct infection of the mucous membrane of the appendix by its contained bacteria.

4. Alterations in its position and shape so as to occlude its lumen, preventing the escape of its natural secretions and contained intestinal contents.

5. Changes in its position and pressure upon its mesentery by growths or impacted feces in the cecum.

6. Alterations in its position or shape so as to shut off its blood supply.

7. Foreign bodies, including fecal concretions.

8. There was one other pathological condition about which the author could obtain no information from the cases tabulated, namely, the abdominal tonsil, referred to by Sutherland, Robinson, Yeo Burney, Haigh, and Bland Sutton.

It is probable that in the present state of surgical knowledge the majority of medical men are in favor of surgical intervention in cases tending towards general peritonitis, even though the symptoms do not point to perforation or abscess formation, in the hope that drainage of the abdominal cavity and the removal of the focus of disease may enable the surgeon to forestall what would otherwise be a fatal complication.

Of 445 cases out of 558 that were examined, 79 per cent. resulted in abscess, perforation, peritonitis, etc.

| | | |
|---|---|---|
| Perforation, without given cause | 11 | |
| With abscess | 10 | |
| With ulceration | 16 | |
| With peritonitis | 32 | |
| With concretion or foreign body | 41 | |
| With gangrene | 7 | |
| With gangrene and concretion | 19 | |
| With inflammation | 13 | |
| With hardened feces | 1 | |
| With concretion and peritonitis | 24 | |
| With foreign body and peritonitis | 7 | —181 |

ABSCESS WITHOUT PERFORATION.

| | | |
|---|---|---|
| Uncomplicated | 136 | |
| Containing concretion | 19 | |
| With perforation | 2 | |
| Sloughing appendix | 2 | |
| Foreign body | 1 | —160 |

| | | |
|---|---|---|
| Foreign bodies | 36 | |
| Fecal concretions | 26 | |
| Inflammation of the appendix | 8 | |
| Enteroliths | 2 | |
| Gangrene of the appendix | 9 | |
| Gangrenous appendix with concretion | 2 | |
| Inflammation with concretion | 3— | 86 |
| GENERAL PERITONITIS. | | |
| With concretion | 5 | |
| Foreign body and gangrenous appendix | 1 | |
| Foreign body | 3 | |
| Ulceration | 1— | 10 |
| Total | | 437 |

It will be noticed that this is a dismal showing for the conservative treatment of appendicitis. The author believes that no case of appendicitis should ever be considered as purely medical. When one is called to a patient, it is his duty to immediately prepare for operative interference. He would advise operation in every case, after studying the subject carefully, where the symptoms showed any tendency to increase. If a tumor was present and the symptoms suggested the possibility of the presence of pus, he would surely operate.

Dr. E. H. Richardson, of Atlanta, followed with a paper on "The Medical Side of Appendicitis." He cited the opinions of eminent medical practitioners, both in this country and Europe, in favor of non-operative interference in a large proportion of cases.

Dr. W. H. Elliott, of Savannah, said the foremost difficulty in cases of appendicitis was to know when and when not to cut. No man was fit to treat appendicitis who was not absolutely willing, at any moment that he might be needed, to open the abdominal cavity.

Dr. Willis F. Westmoreland, of Atlanta, was quite sure that in every case of relapsing appendicitis he had seen, there were adhesions from previous attacks. Some of these cases had died. If they had been operated on in the primary attack, the chances would have been better for their recovery. He would open the abdomen as soon as appendicitis was diagnosed, if the patient would permit it.

Dr. Thos. R. Wright, of Augusta, considered the operation for this disease very difficult, for the reason that no two cases were alike. It was an easy matter to cut down, but if pus and adhesions were found around the appendix the operation was extremely difficult.

Dr. J. W. Duncan, of Atlanta, had seen and treated medicinally several cases of appendicitis during thirty years' practice, one of them having died from a recurrent attack a few months after the primary one. He was satisfied that many cases operated upon were nothing but typhoid fever, and that other cases thought to be typhoid fever were really appendicitis.

Dr. J. B. S. Holmes, of Atlanta, considers the disease a surgical one, and thinks it is safer to operate as soon as a diagnosis is made. The only cases he had lost were those that had been treated medicinally from two to four days previous to operative interference. It an early operation is performed, the mortality should be small.

Dr. Wm. H. Doughty, of Augusta, maintained that it was not prudent to operate upon every case of the disease as soon as the diagnosis was made, for the reason that the surgeon does not always see the cases in their incipiency. If he did, it would be safe to remove the appendix, as the chances were that at this time there would not be pus formation; in other words, the surgeon would have a comparatively clean condition within the abdominal cavity, and the mortality of operations done under these circumstances ought to be small.

Dr. M. L. Boyd, of Savannah, took the position that if all cases could be diagnosed early, it would be wise to operate at once.

Dr. George H. Noble, of Atlanta, thought it was extremely unfortunate that the general practitioner should view appendicitis from a prejudiced standpoint. There were undoubtedly many cases of the catarrhal form that were relieved temporarily, or cured by purgation, possibly permanently. Where we have rupture and the discharge of the contents of the appendix into the peritoneal cavity, we have the fulminant form of the disease, and in such cases very prompt and active measures must be instituted. He advocated early operation.

Dr. L. G. Hardman, of Harmony Grove, said that while there were cases of appendicitis calling for the services of the surgeon, there were others that could be treated successfully by medicinal measures. Of fifteen cases which had come under his care, only two were operated upon. All recovered, both the operative and non-operative cases. In the cases operated on suppuration had occurred, and one of the patients, a physician, was present.

A paper on "Some Albuminuric Complications of Pregnancy" was read by Dr. Howard J. Williams, of Macon. He said this complication was present in from 6 to 50 per cent. of all pregnancies. On the other hand, puerperal eclampsia was comparatively rare. From January 1, 1891, to March 31, 1896, he had encountered ten cases of albuminuria or toxemia in 163 pregnancies, and two cases of eclampsia in this number (ten cases). This gives 6 per cent. of toxemia and about 1 per cent. of eclampsia. The percentage given is based upon accurate analyses of the urine of every woman he had attended during the time mentioned. Albuminuria may be present in varying degrees, from a mere trace to a very large per cent., the urea and other excreta accumulating in the blood in the same proportion. The toxemia is slow or rapid according to the degree of retention of the latter from the outside. These variations were shown by the recital of three cases.

The cardinal indications are to promote elimination of the toxic materials circulating in the blood and to restore the excretory organs to their normal functions. If, however, the poisoning is excessive and the life of the mother and the fetus is at stake, then the indication is to promptly empty the uterus.

Elimination is preëminently indicated for all forms of kidney disease, and in this complication it yields the best results. But if it cannot be tolerated or disagrees, a more liberal diet should be given, such as the white meats of fowls, fish, oysters, fresh fruits, and good, nutritious breads. Remedies to promote elimination by the kidneys were then dwelt upon. Nervous excitement should be controlled by sedatives and narcotics, though not to the extent of interfering with eliminative measures. Elimination of the waste products having failed to restore the functions of the excretory organs and the condition is urgent, eclampsia being imminent or other complications alarming, the uterus should then be emptied.

Dr. R. B. Barron, of Macon, then delivered the "Orator's Address." He dwelt upon and pleaded for higher medical education.

Relative to practicing medicine purely for money, he said that money with the true physician was of secondary consideration; that the real incentive for the best work with those practitioners who achieve success was that broad humanitarianism which impelled with resistless force the commissioned agents of God Almighty to relieve suffering, to stay the pangs of agonizing pain, to fight to the bitter end humanity's implacable and unconquerable enemy, death, regardless of any other consideration.

"The After-Treatment of Tracheotomy Cases of Membranous Croup" was the title of a paper read by Dr. R. M. Harbin, of Rome. (See this issue.)

Dr. J. B. S. Holmes, of Atlanta, reported several interesting surgical cases.

"A Case of Enormous Ventral Hernia Cured by a Plastic or Flap Operation," by George H. Noble, of Atlanta. The subject was a very large woman, who came to him from a neighboring State, giving a meager history, saying that the protrusion first appeared after severe straining, and grew rapidly until it reached the size of an adult head. The treatment she had received consisted in local applications only, no attempt at operative measures having been made. The case is of considerable interest on account of such a large hernia in this region and because expansion of the ribs prevented closure of the ring by approximation of its margins, necessitating, therefore, a plastic or flap operation to close the aperture, which was large enough to pass a closed hand through without resistance.

The operation consisted of: (1) Trimming away the excess of the sac and uniting the peritoneum with buried catgut sutures. (2) Four strong tension sutures were passed through the abdominal walls, piercing the semilunar lines down to the peritoneum, but not implicating it. (3) The semilunar flap was carefully outlined upon either side over the recti muscles with a straight or vertical side upon their outer margins and the convexed borders turned toward and extending to the hernial ring. The aponeurosis of the external oblique and the outer layer of that belonging to the internal oblique muscles were cut through and the flaps liberated except where their bases joined the ring and turned over the opening, accurately abutting the edges, in which position they were stitched with buried silk sutures. The convexed borders coincided with the margins of the ring to which they were made fast. (4) The recti muscles were brought in direct apposition by surrounding them with the large catgut, thus adding another layer of strong tissue over the hernial opening. (5) The skin and fatty tissue were then brought together and the tension sutures tied over all, the wound dressed antiseptically with firm pad, roller bandages, etc. The wound proved entirely aseptic, and the result was very gratifying. In another such case the reader would use buried silver sutures instead of absorbable materials.

Dr. M. B. Hutchins, of Atlanta, read a paper on the "Treatment of Skin Disfigurements by Electrolysis." That electrolysis has a wide application and a legitimate use, he was convinced by his own experience during the past five or six years. To do the work outlined in the paper a few galvanic cells, a sponge electrode, a needle-holder, and for accurate work, a milliamperemeter, are necessary. In most cases the needle should be attached to the negative pole. The current used is weak, and varies from three to seven or eight cells. With a milliamperemeter it is found that we get from one to five milliampères from this current to the relative position of the electrode, the greater amount of intervening tissue reducing the current by resistance, a lesser interval increasing it. The number of needles used, the manner of grasping the sponge electrode, the wetness of the sponge, all influence the strength of the current. The strength of current used in these operations is harmless to the patient, and would not be felt upon the unbroken epidermis.

For the removal of superfluous hairs on the face of ladies, electrolysis was the only method. Many of the patent hair removers were excellent stimulants to its growth. A lounge, good eyes, a good light, a steady hand and perseverance were necessary. Dr. Hutchins had never seen any injury result from the treatment of moles by electrolysis. He had removed one by excision which had become epitheliomatous through a razor cut. There was no recurrence. Moles with hairs in them will frequently disappear from the simple effect of the current used in destroying the hairs, but destroying the mole does not always destroy the hairs, as they are deeper.

Warts of various kinds, so tedious of treatment with the old methods, save that of excision, with or without cauterization, usually dissolve into froth under the action of electrolysis, being more easily destroyed than the average mole. The author then dwelt upon electrolysis in the treatment of epithelioma, small fibromata, milia, sebaceous cysts, keloids and hypertrophic scars, comedones, angiokeratoma, etc.

The paper was based almost entirely upon the author's own practical experience, and his implicit faith in this method of treatment is due both to results in practice and upon himself.

Dr. M. L. Boyd, of Savannah, followed with a report of a few interesting surgical and gynecological cases.

"The Use of Subconjunctival Injections of Mercury in Certain Forms of Eye Diseases," by Dr. Dunbar Roy, of Atlanta. In this paper the author tabulated twenty-five cases of various eye affections, and his experience leads him to the following conclusions: (1) In infected processes of the cornea, ulcers, abscesses, wounds, etc., this method of treatment is quicker and therefore more satisfactory for clinical purposes than those usually employed. (2) In acute iritis it does not seem to exert any beneficial influence, but produces great pain, while in other cases the pain is mitigated. (3) In chronic iritis and iridocyclitis the pain and congestion are often markedly influenced for the better, but it has no effect whatever upon adhesions of the iris to the lens of the capsule. (4) In post-operative infection and panophthalmitis it is by far the best method to which we may resort for good results. (5) In pannus of the cornea absolutely no effect was produced one way or the other. (6) With lesions of the choroid and other deeper structures of the eye he had had no experience, but expects to try this method whenever a case presents itself. From his personal experience he would unhesitatingly say that we cannot depend upon subconjunctival treatment alone, but as an adjuvant it is most excellent, and in some cases produces results far more beneficial than were expected.

Dr. A. W. Stirling, of Atlanta, read a paper on "Glaucoma in Relation to General Practice." He said that glaucoma was to the ophthalmic specialist one of the most interesting of diseases, comprising one per cent. of all ophthalmic troubles. While he believes that every case of the disease requires the most skillful advice that can be obtained, still it is the first and foremost affection which comes within the province of the general practitioner, and with him lies frequently the ultimate safety or destruction of the eye involved. The reader reported several interesting cases, after which he dwelt upon the variability in tension. Coming to the treatment, he counselled against the indiscriminate use of mydriatics, especially atropine, scopolamin, and cocaine in affections of the eye. Glaucoma had often resulted from the use of these agents, and is almost always accentuated by them. In most ocular diseases they are harmful, and the general practitioner would do well to remove them from his list in ophthalmic practice, except when he knows he is dealing with an uncomplicated keratitis or an inflammation of the iris. On the other hand, in myotics, and notably in eserine and pilocarpine, we have a fairly certain means of temporarily benefiting many cases of glaucoma, and of saving valuable time till the most suitable method of treatment can be decided on.

The following officers were elected:

President—Dr. Geo. H. Noble, Atlanta.

First Vice-President—Dr. J. B. Morgan, Augusta.

Second Vice-President—Dr. R. B. Barron, Macon.

Secretary—Dr. R. H. Taylor, Griffin.

Treasurer—Dr. E. C. Goodrich, Augusta.

Place of meeting, Macon, April, 1897.

---

HE—I should think medicine would be a peculiarly difficult profession for a woman. She—Why? He—In order to succeed, she would have to give up trying to look young.—*Puck.*

